# Overburden of informal caregivers of older adults with Post-COVID Syndrome

**DOI:** 10.1590/0034-7167-2025-0184

**Published:** 2026-06-12

**Authors:** Fabiana Carla Matos da Cunha Cintra, Marco Túlio Gualberto Cintra, Daniel Messias Martins, Gabriel Rabelo de Barros Simão, Estevao Alves Valle, Iza Faria-Fortini, Bernardo de Mattos Viana, Maria Aparecida Camargos Bicalho

**Affiliations:** I Universidade Federal de Minas Gerais. Belo Horizonte, Minas Gerais, Brazil; II Clínica Mais 60 Saúde. Belo Horizonte, Minas Gerais, Brazil

**Keywords:** Post-Acute COVID-19 Syndrome, Caregivers, Informal Caregivers, Caregiver Burn, Aged., Síndrome Post Agudo de COVID-19, Cuidadores, Cuidadores Informales, Carga del Cuidador, Anciano.

## Abstract

**Objectives:**

to assess the burden of informal caregivers of older adults with post-COVID syndrome and analyze its association with variables regarding older adults and caregivers.

**Methods:**

a cross-sectional study conducted with 75 older adults with post-COVID syndrome and caregivers. Clinical, functional, cognitive, and psychological measures were used to assess older adults. Psychological and burden measures were used for caregivers using Zarit Burden Interview. Descriptive statistics, Spearman’s correlation, and univariate and multiple linear regression were used.

**Results:**

most caregivers (67%) reported burden, with 16% experiencing high burden, 31% experiencing moderate, and 20% experiencing mild burden. In multivariate analysis, caregiver burden was associated with frailty (p=0.022), older adult depression (p=0.022), and caregiver depression (p<0.001). Variables explained 37% (p<0.001) of variance in the total Zarit Burden Interview score.

**Conclusions:**

caregiver burden for older adults with post-COVID syndrome was associated with factors about older adults and their caregivers, highlighting the need for interdisciplinary support.

## RESUMEN

**Objetivos:**

evaluar la carga de los cuidadores informales de ancianos con síndrome post-COVID y analizar su asociación con variables inherentes a los ancianos y cuidadores.

**Métodos:**

estudio transversal realizado con 75 ancianos con síndrome post-COVID y cuidadores. Utilizamos medidas clínicas, funcionales, cognitivas y psicológicas para evaluar ancianos. Utilizamos medidas psicológicas y de carga para los cuidadores mediante la Zarit Burden Interview. Utilizamos estadísticas descriptivas, correlación de Spearman y regresión lineal univariante y múltiple.

**Resultados:**

la mayoría de los cuidadores (67%) reportaron sobrecarga: el 16% experimentó una sobrecarga alta, el 31% moderada y el 20% leve. En el análisis multivariable, la sobrecarga del cuidador se asoció con fragilidad (p=0,022), depresión en ancianos (p=0,022) y depresión del cuidador (p<0,001). Estas variables explicaron el 37% (p<0,001) de la varianza en la puntuación total de la Zarit Burden Interview.

**Conclusiones:**

la sobrecarga del cuidador en ancianos con síndrome post-COVID se asoció con factores relacionados con el adulto mayor y su cuidador, lo que resalta la necesidad de apoyo interdisciplinario.

## INTRODUCTION

Five years after the start of the COVID-19 pandemic, millions of people continue to suffer from long-term consequences of SARS-CoV-2 infection. Post-COVID-19 symptoms, referred to as long COVID or post-COVID syndrome (PCS), can be defined as a heterogeneous disorder that occurs in individuals with a history of probable or confirmed SARS-CoV-2 infection, typically manifesting three months after the onset of COVID-19, with symptoms lasting at least two months and not explained by an alternative diagnosis^(1,2)^. In addition to acute complications caused to the respiratory system, previous studies report the occurrence of chronic symptoms, such as fatigue, dyspnea, cognitive changes, anxiety, and depression, which can become debilitating and affect the daily routine of affected individuals^(1,3)^.

Since the beginning of the COVID-19 pandemic, older adults have been the most affected group. Frail older adults accounted for approximately 51% of hospitalized patients and presented a higher risk of mortality compared to non-frail older adults^(4)^. The additional burden on caregivers of older adults with COVID-19 can compromise the quality of informal care, preventing older adults from receiving care safely in their homes and, consequently, increasing the risk of unfavorable outcomes, such as increased demand for emergency services, hospitalization, or long-term institutionalization^(5)^. Among Japanese informal caregivers, 41% reported increased burden after the pandemic^(6)^. However, this assessment was conducted online, and overload was assessed using an informal, non-standardized question^(6)^. According to a qualitative systematic review published in 2022, although there are important limitations in the studies, it is possible to conclude that the COVID-19 pandemic increased the burden on informal caregivers^(7)^.

In Brazil, family responsibility and the burden on the healthcare system regarding the care of older adults have increased with the COVID-19 pandemic^(8)^. It is worth noting that the family represents the main source of assistance for older adults^(8)^. The physical and emotional overload experienced by caregivers of older adults has been associated with factors such as perceived effort, older adults’ dependence, caregiver age, symptoms of emotional overload, and low income^(9)^. However, considering the particularities of PCS symptoms in older adults, it is necessary to investigate whether there are any changes in the factors related to the burden of caregivers of older adults after COVID-19 diagnosis^(6)^.

Although the consequences of PCS are a widely researched topic, studies that consider a comprehensive, in-person assessment of older adults and their caregivers after a COVID-19 diagnosis are still scarce. This topic lacks analysis in the literature, especially for older adults living in lowand middle-income countries, such as Brazil^(1)^. Identifying the burden on caregivers of older adults with PCS is extremely important for developing better care conditions for the caregiver-older adult dyad and for directing long-term healthcare services^(8,9)^.

## OBJECTIVES

To assess the burden of informal caregivers of older adults with PCS and analyze its association with sociodemographic, clinical, cognitive, and psychological variables inherent to older adults and their caregivers.

## METHODS

### Ethical aspects

The study was conducted in accordance with national and international ethics guidelines, and was approved by the *Universidade Federal de Minas Gerais* (UFMG) Research Ethics Committee. All participants and their guardians were informed of the research objectives and data confidentiality, and consented to participate by signing the Informed Consent Form (ICF).

### Study design, period and site

This is an observational, analytical, cross-sectional study, based on a study sample approved by the UFMG Research Ethics Committee. The study was conducted between July 2021 and February 2025 and involved home or outpatient care at *the Instituto Jenny de Andrade Faria* (IJAF), which provides healthcare for older adults and women at the UFMG *Hospital das Clínicas* (UFMG-HC), and at the *Mais 60 Saúde* clinic. Participants aged 60 years or older were recruited from individuals discharged from hospitals due to COVID-19 at two general hospitals in Belo Horizonte, Minas Gerais, UFMG-HC and Hospital Luxemburgo, or from those treated at the *Mais 60 Saúde* clinic in Belo Horizonte, a geriatric and gerontological outpatient care unit, or from older adults in the community.

This article was written according to SRrengthening the Reporting of OBservational studies in Epidemiology guidelines^(10)^.

### Sample and inclusion and exclusion criteria

The study was conducted with a non-probabilistic, consecutive sample of older adults with PCS and their respective caregivers, recruited from the community and from the hospital and outpatient settings described above through active search, invitations posted on social media, or contact lists provided by partner institutions after subjects’ hospital discharge. Sample size calculation assumed a power of 90% and a mean standard error of 5%. Based on these parameters, the minimum sample size required was estimated at 56 older adults and their respective caregivers.

PCS was characterized by the presence of symptoms lasting at least two months, occurring three months or more after COVID-19 diagnosis, not explained by another alternative diagnosis^(2)^.

Inclusion criteria comprised subjects aged 60 or older after initial COVID-19 diagnosis, confirmed by laboratory testing, with diagnostic criteria for PCS, and the presence or indication of contact with an informal caregiver. Caregivers needed to be familiar with older adults’ daily routine and be over 18 years of age. Exclusion criteria included family members or caregivers with severe mental disorders who did not demonstrate understanding of the proposed questions; older adults with serious health conditions without medical control; those with active cancer; dementia prior to the COVID-19 diagnosis; institutionalized older adults; those with severe mobility or communication deficits that prevented completion of the assessment protocol; and those who refused to participate in the study or did not sign the informed consent form.

### Study protocol

After older adults were screened by phone, an initial in-person appointment was scheduled at the post-COVID outpatient clinic at IJAF, at the *Mais 60 Saúde* clinic, or at their home. Participants underwent an interview and followed the study protocol, specifically developed by the researchers for assessing older adults and their caregivers. The protocol included sociodemographic data and standardized measures of clinical, psychological, functional, and cognitive assessment, widely used in research and clinical practice, and culturally adapted for the Brazilian context.

Participants underwent an initial medical assessment, as per the study’s care protocol, including a physical examination, blood draws, and laboratory and neuroimaging tests. After this stage, they continued with the study’s assessment protocol, which lasted approximately two hours.

Selected subjects who met inclusion criteria were invited to participate in the study and signed the informed consent form. They underwent three follow-up assessments: an initial assessment up to 24 months after the initial infection, called AS1; another, called AS2, after six months; and a third assessment (AS3), 12 months after the date of the first assessment. For this study, only the data from AS1 were analyzed. All assessed cases were discussed with the outpatient geriatrician-preceptor and an occupational therapist, both with over 15 years of training and experience in geriatrics and gerontology, respectively.

To characterize the sample, sociodemographic data (age, gender, education, degree of kinship, income, who lived with the older adult, and property ownership) were used. To assess caregivers, the Zarit Caregiver Burden Interview (ZBI) and the Hamilton Rating Scale for Depression (HAM-D) were used. The instruments used to assess the older adult, selected from an extensive literature review on PCS symptoms in older adults^(1,3,4)^, are described below:

### Clinical measures

The Clinical-Functional Vulnerability Index-20 (In Portuguese, *Índice de Vulnerabilidade Clínico-Funcional-20* - IVCF-20) was used to define older adults at risk of frailty, with a cut-off score of 7-14 points, and frail older adults, with a cut-off score ≥15 points^(11)^. To assess the level of prior and acquired frailty after COVID-19 diagnosis, the Clinical Frailty Scale (CFS) was used, with a cut-off score of ≥5 points for the presence of frailty^(12)^.

The Modified Medical Research Council (mMRC) scale, rated from 0 to 4, was used to measure the severity of dyspnea^(13)^. The Fatigue Severity Scale (FSS) was used to measure the intensity of fatigue and its impact on older adults’ life and activities. The cut-off score was ≥28 points^(14)^.

### Functional measures

The Alzheimer’s Disease Cooperative Study (ADCS) - Activities of Daily Living, Brazilian version, assesses the ability of older adults with cognitive impairment to perform basic, instrumental, and advanced activities of daily living. CINTRA *et al*. (2017)^(15)^ proposed that a score <72 points was suggestive of functional decline in older adults with mild cognitive impairment, and a score ≤65 points was suggestive of dementia with significant functional impairment^(15)^.

The Functional Independence Measure (FIM) scale was used to assess older adults’ performance in self-care, sphincter control, transfers, locomotion, communication, and social cognition. There is no cut-off score, but a score below 126 points suggests functional impairment^(16)^.

### Cognitive measures

The Dementia Screening Interview (AD8) cognitive screening test was used, and a score ≥2 was considered suggestive of cognitive impairment^(17)^.

The Z-score of the sum of points obtained from the total points of the Mattis Dementia Rating Scale (MDRS)^(18)^ adjusted for age and education was used to assess the presence of cognitive impairment.

Cognitive diagnosis was defined through discussion between a geriatrician and an occupational therapist with training and experience in gerontology, through analysis of the MDRS adjusted for age and education and the ADCS.

### Psychological measures

Diagnostic and Statistical Manual of Mental Disorders - 5^th^ edition (DSM-5) criteria were used, with a cut-off of ≥5 criteria^(19)^, for major depressive disorder.

A score ≥44 points on the Post-Traumatic Stress Disorder Checklist - Civilian Version (PCL-C)^(20)^ was used to diagnose post-traumatic stress disorder (PTSD) in older adults.

### Caregiver assessment

The ZBI was applied to assess the burden of caregivers of older adults, classified as no burden (score less than or equal to 15 points), mild burden (score between 16 and 22), moderate burden (score between 23 and 33), and high burden (score greater than or equal to 34)^(21)^.

The HAM-D was used to assess the severity of depressive symptoms. A cut-off point of >7 points was used as a suggestive indicator of depressive symptoms^(22)^.

### Analysis of results and statistics

Initially, the Shapiro-Wilk normality test was used. Numerical variables were presented using descriptive statistics, with mean, median, and standard deviation being calculated, and categorical variables were presented using relative and absolute frequencies.

Since the data were not normally distributed, except for caregiver age, Spearman’s correlation test was used to assess correlations between the dependent variable (caregiver burden) and independent variables such as functionality, frailty, cognition, mood, dyspnea, fatigue, post-traumatic stress, and sociodemographic factors. Caregiver burden was adopted as a continuous variable, represented by the total ZBI score, and independent variables were selected from a literature review on PCS symptoms in older adults.

Linear regression analysis was used to estimate how much each independent variable contributed to the variation in the total ZBI score. In univariate analysis, variables were assessed individually, and those with a p-value <0.20 were entered into the final multiple linear regression analysis, using the backward method to adjust for potential confounders. In this method, all selected variables were entered simultaneously into the model, and then new models were estimated by removing each variable based on the highest p-value.

Statistical analyses were performed using Jeffreys’s Amazing Statistics Program (Version 0.19.1.0). The significance level adopted for all analyses was 5% or p<0.05.

## RESULTS

The sample consisted of 75 older adult participants with PCS and their respective caregivers, after an average period of 10.09±5.97 (Min.=2; Max.=24) months between COVID-19 diagnosis and AS1.

Regarding caregivers of older adults with PCS, the study showed that 77.92% were female, with a mean age of 54.99 ± 15.10 years and 10.75 ± 5.01 years of education. Of these, 46.75% were children; 37.66% were spouses; 10.39% were friends; and 5.20% were siblings. Concerning caregivers’ income, 55.84% did not work formally, and 46.75% earned one to two minimum wages. It is noteworthy that the majority (84.62%) of older adults owned their own home and that 58.44% of caregivers lived with older adults.

**Figure 1:**
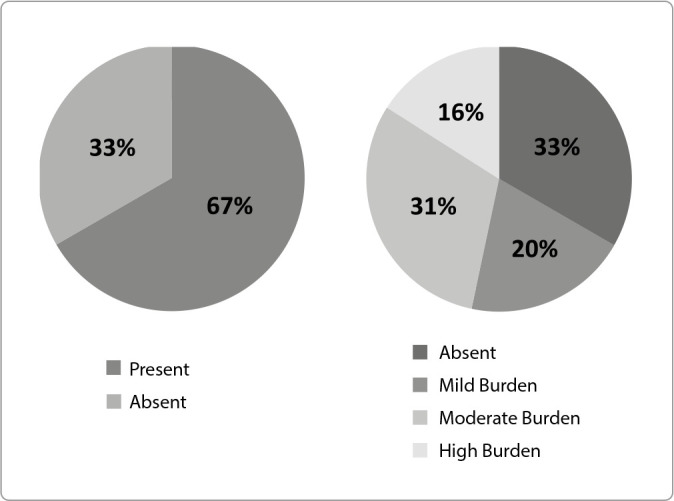
Prevalence and intensity of caregiver burden for older adults with post-COVID syndrome

The HAM-D assessment showed that 44.60% of caregivers presented depressive symptoms, with a mean score of 7.49 ± 5.98 points. Among those depressed, 31.51% had mild intensity; 33.51% had moderate intensity; and 34.98% had high intensity. Caregiver burden was present in 67% of the sample, and of these, 33% had moderate intensity, as shown in Figure 1. The mean ZBI was 22.81 ± 13.41 points.

**Figure 2:**
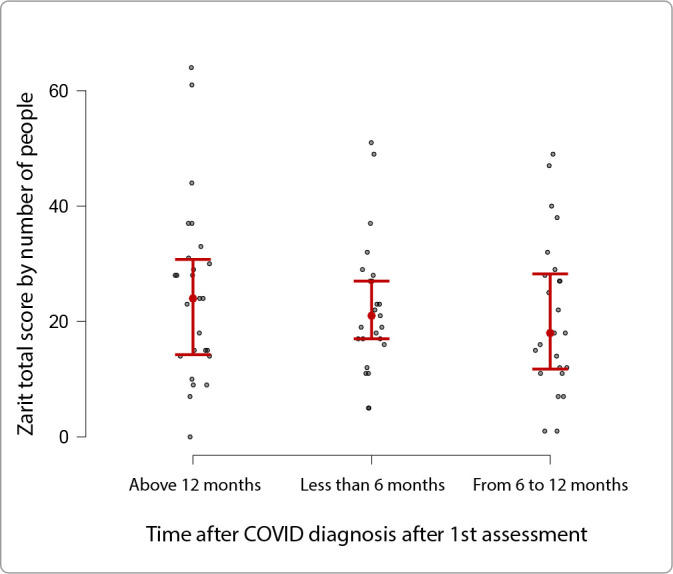
Caregiver burden relative to time since COVID-19 diagnosis and date of first assessment

The presence of caregiver burden remained constant among participants, even among those with a different time interval between COVID-19 diagnosis and assessment (p=0.675), as shown in Figure 2.

In relation to the characteristics of older adults under care, 51.25% were female, with a mean age of 73.91 ± 10.20 years, 8.03 ± 5.78 years of education, 1.79 ± 1.79 clinical comorbidities, polypharmacy (6.54 ± 5.00 medications in use), and 3.39 ± 1.63 doses of COVID-19 vaccine at the time of initial infection. Furthermore, 57.50% were hospitalized; 15% were admitted to the Intensive Care Unit; and 70.87% self-reported memory difficulties after COVID-19.

Regarding cognitive capacity at the time of the first assessment, the majority (77.5%) of older adults presented cognitive impairment, with 62.5% having mild cognitive impairment, 15% having dementia, and 22.5% having normal cognition. Self-reported frailty assessment, according to the previous CFS (one month before the COVID-19 diagnosis), indicated that 26.25% of older adults were frail. In the first assessment, a significant increase in acquired frailty was observed after COVID-19, with 47.50% of older adults being frail (p<0.001), when compared to the self-reported previous CFS.

Table 1 shows the description of clinical, cognitive, psychological and functional measures of the older adults participating in this study:

**Table 1:** Description of clinical, cognitive, psychological, and functional measures of the older adults assessed, Belo Horizonte, Minas Gerais, Brazil, 2025

Variable	Measuring instrument	Mean/standard deviation
Prior frailty	CFS	3.63±1.32
Post-COVID frailty	CFS	4.25±1.36
Frailty	IVCF-20	12.93±7.44
Fatigue	FSS	29.68±17.62
Dyspnea	mMRC	2.18±1.31
Post-traumatic stress disorder	PCL-C	29.08±11.26
Older adult depression	DSM-5	1.78±2.14
Functionality	ADCS-AVD	63.91±11.21
Functionality	FIM	111.23±11.62
Cognition	AD8	2.89±2.01
Cognition	Score Z MDRS	-2.40±3.62

*CFS - Clinical Frailty Scale; IVCF-20 - Índice de Vulnerabilidade Clínico Funcional-20; FSS - Fatigue Severity Scale; mMRC - Modified Medical Research Council; PCL-C - Post-Traumatic Stress Disorder Checklist - Civilian Version; DSM-5 - Diagnostic and Statistical Manual of Mental Disorders - 5th edition; ADCS-AVD - Alzheimer’s Disease Cooperative Study - Activities of Daily Living; FIM - Functional Independence Measure; AD8 - Dementia Screening Interview; MDRS - Score Z Mattis Dementia Rating Scale.*

The caregiver burden of older adults with PCS correlated with the following variables: caregiver depressive symptoms, assessed by HAM-D (rho=0.523; p-value<0.001); post-COVID frailty of older adult patients, measured by IVCF-20 (rho=0.347; p=0.002) and CFS (rho=0.385; p<0.001); PTSD of older adult patients (rho=0.311; p=0.007); depressive symptoms of older adult patients, measured by DSM-5 (rho=0.345; p=0.002); functional disability of older adult patients, measured by ADCS (rho=-0.358; p=0.002) and FIM (rho=-0.364; p=0.001); frailty prior to COVID-19 in older adult patients, measured by CFS (rho=0.267; p=0.020); and cognitive decline in older adult patients post-COVID, assessed by AD8 (rho=0.294; p=0.011).

Variables with a p-value <0.020 were selected for multiple linear regression analysis. Multivariate analysis indicated positive and significant coefficients for older adult frailty after COVID-19 (CFS; p=0.022), older adult depression (DSM-5; p=0.022), and caregiver depression (HAM-D; p<0.001), suggesting that an increase in these variables is associated with an increase in the ZBI. Together, these factors explain 37% of the variance in the total ZBI score, with adjusted R2=0.370.

## DISCUSSION

The results of this study indicate that most caregivers of older adults experience moderate burden after a COVID-19 diagnosis. The main determinants were older adults’ post-COVID-19 frailty and depression in both the older adult and caregiver, which together explained 37% of older adults’ post-COVID-19 caregiver burden, as measured by the ZBI.

As a result of the pandemic, caregivers of older adults, especially female caregivers, have become more likely to experience an increase in the intensity of care burden and a decline in self-perceived health^(23)^. These data support the current study results, as well as another Brazilian study published in 2022, which describes the burden, depressive symptoms and the profile of informal caregivers of older adults during the COVID-19 pandemic^(24)^.

A study published in 2022, which explored factors associated with increased caregiver burden during the pandemic, observed an association between depressive symptoms in caregivers and higher care burden scores (OR=2.20; 95%CI, 1.50-3.23), a result similar to that of this study^(6)^. In addition to caregiver depressive symptoms, other factors were also associated with caregiver burden, such as low Barthel Index scores (OR=2.01, 95%CI, 1.39-2.90), presence of a dementia diagnosis (OR=2.48, 95%CI, 1.07-5.73), length of caregiving (OR=1.09, 95%CI, 1.01-1.17), and use of home care services (OR=1.46; 95%CI, 1.01-2.10)^(7)^. However, they differ from ours, probably due to the sample characteristics, the difference in the assessment instruments used, and the time between diagnosis and assessment.

Another study, published in 2021, investigated factors associated with depressive symptoms in caregivers of older adults diagnosed with COVID-19 during the pandemic in Serbia, and found results similar to this study^(25)^. The authors observed that 71.9% of informal caregivers experienced care burden, and 27.1% of them presented depressive symptoms. Self-rated physical health, the need for psychosocial support, and caregiver burden were the main direct predictors of caregiver depression. The authors suggest that self-reported burden is a significant risk factor for depressive symptoms in caregivers of older adults with COVID-19 during the pandemic^(25)^.

Caring for a frail older adult is a dynamic process that can also weaken caregivers, who may feel powerless in the face of comprehensive needs, since older adults’ frailty is generally associated with unfavorable clinical conditions, such as susceptibility to diseases, functional decline, falls, hospitalization, and early death^(26)^. Furthermore, prolonged social isolation, intensified by the pandemic, combined with the presence of multiple chronic diseases, can exacerbate mental health problems, such as depression and anxiety, in older adults^(6,9,24)^.

Additionally, caregivers of older adults are recognized as a psychologically vulnerable group, especially when their own needs are neglected^(25)^. The pandemic has exacerbated limitations on access to formal healthcare services, already characterized by insufficient structural and coverage, forcing informal caregivers to take on additional responsibilities in more adverse conditions^(25)^. This combination of factors requires caregivers to provide constant assistance, which can lead to excessive stress, both physical and emotional^(6,9,24)^.

A study carried out in Minas Gerais in 2022 showed that, during the pandemic, older adults evolved with greater dependence on instrumental activities of daily living, as well as worsening cognition^(27)^, which was accompanied by a decrease in families’ availability to meet this new demand^(28,29)^.

Although previous studies have addressed additional factors, such as cognitive and functional disability, associated with the burden of family caregivers of older adults during the COVID-19 pandemic and of older adults in the community^(30,31)^, in the present study, we did not observe a correlation between these variables and the burden of caregivers of older adults after COVID-19 infection in the multivariate analysis. A plausible explanation would be that persistent PCS symptoms possibly impact more complex activities of daily living, such as instrumental and advanced activities, implying less demand for physical care for family caregivers, when compared to the impairment of basic activities of daily living^(32)^. It should be emphasized that these activities, especially social participation, demonstrate a successful aging process^(33)^, and are precursors to a further future decline in activities of less cognitive complexity, such as self-care^(34)^. Furthermore, long-term care actions become repetitive, which may lead caregivers to develop adaptive strategies, such as seeking help from other individuals, both to develop the care activities required by older adults and for themselves, especially to temporarily disengage from this role^(28,31)^.

Evidence suggests that caring for a frail, depressed, and dependent patient after a COVID-19 diagnosis can be a traumatic and negative experience for caregivers, leading to the development of emotional stress and increased intensity of burden, as caregivers need to gradually take on more tasks over the long term^(5,24)^.

Finally, the results of this study point to the urgent need to expand and strengthen public policies aimed at older adults and their caregivers, after COVID-19 diagnosis, in their different areas of care^(29)^.

The need for future studies, especially longitudinal studies, is highlighted to establish risk factors and assess the evolution of care burden in older adults with PCS.

### Study limitations

This study has some limitations, such as its single-center sample and the fact that it was conducted mostly with older adults previously hospitalized for COVID-19, which may hinder generalization of results. The study lacks a control group, and there is a lack of pre-COVID-19 data, especially regarding the presence of burden, which was not assessed retrospectively to avoid recall bias among participants due to its subjective nature. Furthermore, the length of time the informal caregiver spent with the older adult was not assessed, which may influence, at least in part, the burden results. This study, although using standardized measures, uses self-reported questionnaires that may be subject to recall and information bias related to older adults’ and caregivers’ physical and psychological conditions. Additionally, a significant increase in acquired frailty after COVID-19 was observed. However, the development of morbidities, hospital readmissions, and other complications in the period between COVID-19 diagnosis and protocol implementation may have contributed to the increase in frailty in the population studied.

Finally, the design of this study does not allow the identification of risk factors, and may not have analyzed other factors that could possibly be related to care burden in this population.

### Contributions to health

This is one of the few studies that aimed to investigate, through comprehensive, in-person geriatric assessment, the prevalence and factors associated with caregiver burden for older adults with PCS. The results of this study can help guide interdisciplinary team interventions for this population, as they identify factors that can be prevented, controlled, and rehabilitated.

## CONCLUSIONS

The findings of this study demonstrate the occurrence of burden in caregivers of older adults with PCS. This condition was of moderate intensity, positively associated with older adults’ frailty and depressive symptoms in both the older adult and the caregiver.

By identifying the occurrence and factors associated with the burden of caregivers of older adults with PCS, the present study provides significant information and has important implications for clinical practice, as it reveals indicators, suggesting that healthcare professionals should be aware of the caregiver-older adult dyad.

## Data Availability

The research data are available only upon request.
