# Supplementary material for: Overburden of informal caregivers of older adults with Post-COVID Syndrome
Source: Rev Bras Enferm. 2026 Jun 12;79:e20250184. doi: 10.1590/0034-7167-2025-0184 (PMC13262877; doi:10.1590/0034-7167-2025-0184)
Supplement: Translation

# Translation: Sobrecarga de cuidadores informais de idosos com Síndrome Pós-COVID

**DOI:** 10.1590/0034-7167-2025-0184pt

**Authors:** Fabiana Carla Matos da Cunha Cintra, Marco Túlio Gualberto Cintra, Daniel Messias Martins, Gabriel Rabelo de Barros Simão, Estevao Alves Valle, Iza Faria-Fortini, Bernardo de Mattos Viana, Maria Aparecida Camargos Bicalho

**Affiliations:** I Universidade Federal de Minas Gerais. Belo Horizonte, Minas Gerais, Brasil; II Clínica Mais 60 Saúde. Belo Horizonte, Minas Gerais, Brasil

**Keywords:** Síndrome Pós-COVID-19 Aguda, Cuidadores, Cuidadores Informais, Sobrecarga do Cuidador, Idoso.

## Abstract

**Objetivos:**

avaliar sobrecarga de cuidadores informais de idosos com síndrome pós-COVID e analisar sua associação com variáveis inerentes aos idosos e seus cuidadores.

**Métodos:**

estudo transversal, realizado com 75 idosos com síndrome pós-COVID e seus respectivos cuidadores. Para avaliação do idoso, utilizaram-se medidas clínicas, funcionais, cognitivas e psicológicas. Para o cuidador, utilizaram-se medidas psicológicas e de sobrecarga, mensuradas pela *Zarit Burden Interview*. Empregaram-se estatística descritiva, correlação de Spearman, regressão linear univariada e múltipla.

**Resultados:**

a maioria dos cuidadores (67%) reportou sobrecarga, sendo 16% com sobrecarga alta, 31%, moderada, e 20%, leve. Na análise multivariada, a sobrecarga do cuidador associou-se à fragilidade (p=0,022), depressão do idoso (p=0,022) e depressão do cuidador (p<0,001). Essas variáveis explicam 37% (p<0,001) da variância no escore total da *Zarit Burden Interview*.

**Conclusões:**

a sobrecarga do cuidador de idosos com síndrome pós-COVID foi associada a fatores relacionados ao idoso e seu cuidador, evidenciando necessidade do apoio interdisciplinar.

## INTRODUÇÃO

Cinco anos após o início da pandemia de COVID-19, milhões de pessoas continuam sofrendo com sequelas de longo prazo da infecção por SARS-CoV-2. Os sintomas pós-COVID-19, denominados por COVID longa ou síndrome pós-COVID (SPC), podem ser definidos como um distúrbio heterogêneo que ocorre em indivíduos com histórico de infecção provável ou confirmada por SARS-CoV-2, manifestando-se, tipicamente, três meses após o início da COVID-19, com sintomas que duram pelo menos dois meses e não são explicados por um diagnóstico alternativo^(1,2)^. Além das complicações agudas ocasionadas ao sistema respiratório, estudos prévios reportam a ocorrência de sintomas crônicos, como fadiga, dispneia, alterações cognitivas, ansiedade e depressão, que podem se tornar debilitantes e afetar a rotina diária dos indivíduos acometidos^(1,3)^.

Desde o início da pandemia de COVID-19, os idosos têm sido o grupo mais afetado. Os idosos frágeis foram aproximadamente 51% dos pacientes hospitalizados, além de apresentarem um risco elevado de mortalidade em comparação aos idosos sem fragilidade^(4)^. A sobrecarga adicional do cuidador de idosos com COVID-19 pode comprometer a qualidade dos cuidados informais, impedindo que os idosos recebam cuidados com segurança em suas casas e, consequentemente, aumentando o risco de desfechos desfavoráveis, como a maior procura por serviços de emergência, hospitalização ou institucionalização a longo prazo^(5)^. Entre cuidadores informais japoneses, observou-se que 41% relataram aumento da sobrecarga após a pandemia^(6)^. Entretanto, essa avaliação foi conduzida no formato *online*, e a sobrecarga foi avaliada a partir de uma pergunta informal, não padronizada^(6)^. De acordo com revisão sistemática qualitativa, publicada em 2022, embora existam limitações importantes nos estudos, é possível concluir que a pandemia de COVID-19 elevou a sobrecarga do cuidador informal^(7)^.

No Brasil, a responsabilidade da família e a carga sobre o sistema de saúde em relação aos cuidados de idosos aumentaram com a pandemia de COVID-19^(8)^. Destaca-se que a família representa a principal fonte de assistência à população idosa^(8)^. A sobrecarga física e emocional apresentada por cuidadores de pessoas idosas tem sido associada a fatores como percepção do esforço, dependência do idoso, idade do cuidador, sintomas de sobrecarga emocional e baixa renda^(9)^. Todavia, considerando as particularidades dos sintomas da SPC em pessoas idosas, é preciso investigar se há alguma modificação nos fatores relacionados à sobrecarga de cuidadores de idosos após o diagnóstico de COVID-19^(6)^.

Embora as consequências da SPC seja um tema muito investigado, estudos que consideram uma avaliação ampla e presencial de idosos e seus cuidadores após o diagnóstico de COVID-19 ainda são escassos. Esse tema carece de análises na literatura, sobretudo em idosos que residem em países de baixa e média renda, como o Brasil^(1)^. A identificação da sobrecarga em cuidadores de idosos com SPC é extremamente importante para o desenvolvimento de melhores condições de cuidado do binômio cuidador-idoso e para o direcionamento dos serviços de saúde a longo prazo^(8,9)^.

## OBJETIVOS

Avaliar a sobrecarga de cuidadores informais de idosos com SPC e analisar sua associação com variáveis sociodemográficas, clínicas, cognitivas e psicológicas inerentes aos idosos e seus cuidadores.

## MÉTODOS

### Aspectos éticos

O estudo foi conduzido conforme as diretrizes de ética nacionais e internacionais, sendo aprovado pelo Comitê de Ética em Pesquisa da Universidade Federal de Minas Gerais (UFMG). Todos os participantes e seus responsáveis foram informados sobre os objetivos da pesquisa e a confidencialidade dos dados, consentindo com a participação por meio da assinatura do Termo de Consentimento Livre e Esclarecido (TCLE).

### Desenho, período e local do estudo

Trata-se de estudo observacional, analítico e transversal, realizado a partir de recorte do estudo aprovado pelo Comitê de Ética em Pesquisa da UFMG. O estudo foi desenvolvido entre julho de 2021 e fevereiro de 2025, e envolveu atendimento domiciliar ou ambulatorial no Instituto Jenny de Andrade Faria (IJAF) de atenção à saúde do idoso e da mulher do Hospital das Clínicas da UFMG (HC-UFMG) e na clínica Mais 60 Saúde. Participantes com idade maior ou igual a 60 anos foram recrutados entre egressos de internação hospitalar por COVID-19 em dois hospitais gerais de Belo Horizonte, Minas Gerais, o HC-UFMG e o Hospital Luxemburgo, ou entre os atendidos na clínica Mais 60 Saúde, em Belo Horizonte, uma unidade de atendimento ambulatorial geriátrico e gerontológico ou entre idosos na comunidade.

O presente artigo foi redigido segundo as diretrizes do *STrengthening the Reporting of OBservational studies in Epidemiology* ^(10)^.

### Amostra e critérios de inclusão e exclusão

O estudo foi realizado com uma amostra não probabilística e consecutiva composta por idosos com SPC e seus respectivos cuidadores, recrutados na comunidade e nos ambientes hospitalar e ambulatorial descritos anteriormente por meio de busca ativa, convite divulgado em redes sociais ou obtenção de lista de contatos fornecida por instituições parceiras após a alta hospitalar dos sujeitos. Para o cálculo amostral, consideraram-se um poder de análise de 90% e erro padrão médio de 5%. Assim, a partir desses parâmetros, a amostra mínima necessária foi estimada em 56 idosos e seus respectivos cuidadores.

A SPC foi caracterizada pela presença de sintomas com duração de pelo menos dois meses, com ocorrência três meses ou mais após o diagnóstico de COVID-19, não explicada por outro diagnóstico alternativo^(2)^.

Os critérios de inclusão foram ser sujeitos com 60 anos ou mais após o diagnóstico inicial de COVID-19, confirmado por teste laboratorial, com critérios diagnósticos de SPC e a presença ou indicação de contato de um cuidador informal. Para o cuidador, era necessário conhecer a rotina diária do idosos e ter idade superior a 18 anos. Os critérios de exclusão foram familiar ou cuidador que apresentasse transtorno mental grave e não demonstrasse compreensão das questões propostas; idosos com condições graves de saúde e sem controle médico; com câncer ativo; demência prévia ao diagnóstico de COVID-19; idosos institucionalizados; com déficits graves de mobilidade ou comunicação que impedissem o preenchimento do protocolo de avaliação; e aqueles que se recusaram a participar do estudo ou não assinaram o TCLE.

### Protocolo do estudo

Após a triagem dos idosos por telefone, era agendada uma consulta inicial e presencial no ambulatório pós-COVID localizado no IJAF, na clínica Mais 60 Saúde ou no domicílio. Os sujeitos foram submetidos à entrevista e ao protocolo do estudo, elaborado especificamente pelos pesquisadores para avaliação do idoso e de seu cuidador. O protocolo incluiu dados sociodemográficos e medidas padronizadas de avaliação clínica, psicológica, da funcionalidade e cognição, amplamente utilizadas em pesquisa e na prática clínica, adaptadas culturalmente para o contexto brasileiro.

Os participantes foram submetidos à avaliação médica inicial, conforme o protocolo assistencial do estudo, incluindo exame físico, coleta de sangue, e solicitação de exames laboratoriais e de neuroimagem. Após essa etapa, eles prosseguiram com o protocolo de avaliação do estudo, com duração aproximada de duas horas.

Os sujeitos selecionados que preencheram os critérios de inclusão foram convidados a participar do estudo e a assinar o TCLE. Eles foram submetidos a três avaliações de seguimento: uma avaliação inicial até 24 meses da infecção inicial, denominada (AV1); outra denominada (AV2), após seis meses; e uma terceira avaliação (AV3), 12 meses após a data da primeira avaliação. Para este presente estudo, somente os dados da (AV1) foram analisados. Todos os casos avaliados foram discutidos com o médico geriatra preceptor do ambulatório e com uma terapeuta ocupacional, ambos com mais de 15 anos de formação e experiência em geriatria e gerontologia, respectivamente.

Para caracterização da amostra, foram utilizados dados sociodemográficos (idade, gênero, escolaridade, grau de parentesco, renda, quem residia com o idoso e posse do imóvel), e para avaliação do cuidador, utilizaram-se a *Zarit Caregiver Burden Interview* (ZBI) e a *Hamilton Rating Scale for Depression* (HAM-D). Os instrumentos utilizados para avaliação do idoso, selecionados a partir de extensa revisão de literatura acerca dos sintomas da SPC em pessoas idosas^(1,3,4)^, são descritos a seguir:

### Medidas clínicas

O Índice de Vulnerabilidade Clínico-Funcional-20 (IVCF-20) foi utilizado para definir o idoso em risco de fragilização, com nota de corte de 7-14 pontos, e o idoso frágil, com nota de corte ≥15 pontos^(11)^. Para avaliar o nível de fragilidade prévia e adquirida após o diagnóstico de COVID-19, foi empregada a *Clinical Frailty Scale* (CFS), com nota de corte ≥5 pontos para a presença de fragilidade^(12)^.

A escala *Modified Medical Research Council* (mMRC), classificada de 0 a 4, foi utilizada para mensurar a gravidade da dispneia^(13)^. A *Fatigue Severity Scale* (FSS) foi empregada para mensurar a intensidade da fadiga e o seu impacto na vida e nas atividades do idoso. A nota de corte foi ≥28 pontos^(14)^.

### Medidas funcionais

A *Alzheimer’s Disease Cooperative Study* (ADCS) - *Activities of Daily Living*, versão brasileira, avalia a capacidade de idosos com comprometimento cognitivo de realizar atividades diárias básicas, instrumentais e avançadas. CINTRA *et al*. (2017)^(15)^ propuseram que uma pontuação <72 pontos era sugestiva de declínio funcional em idosos, com comprometimento cognitivo leve, e uma pontuação ≤65 pontos era sugestiva de demência, com comprometimento funcional significativo^(15)^.

A escala Medida de Independência Funcional (MIF) foi utilizada para avaliar o desempenho do idoso em autocuidados, controle de esfíncteres, transferências, locomoção, comunicação e cognição social. Não há uma nota de corte, mas uma pontuação abaixo de 126 pontos é sugestiva de comprometimento funcional^(16)^.

### Medidas cognitivas

O teste de triagem cognitiva *Dementia Screening Interview* (AD8) foi utilizado, e uma pontuação ≥2 foi considerada sugestiva de comprometimento cognitivo^(17)^.

O escore Z da soma dos pontos obtidos do total de pontos da *Mattis Dementia Rating Scale* (MDRS)^(18)^ ajustados para idade e escolaridade foi utilizado para avaliação da presença de comprometimento cognitivo.

O diagnóstico cognitivo foi definido por meio de discussão entre geriatra e terapeuta ocupacional com formação e experiência em gerontologia, por meio da análise da MDRS ajustada para idade e escolaridade e da ADCS.

### Medidas psicológicas

Foram utilizados os critérios do *Diagnostic and Statistical Manual of Mental Disorders - 5^th^ edition* (DSM-5), com corte ≥5 critérios^(19)^, para transtorno depressivo maior.

Pontuação ≥44 pontos na *Post-Traumatic Stress Disorder Checklist - Civilian Version* (PCL-C)^(20)^ foi empregada para o diagnóstico de transtorno de estresse pós-traumático (TEPT) do idoso.

### Avaliação do cuidador

A ZBI foi aplicada para avaliação de sobrecarga de cuidadores de idosos, classificada como ausência de sobrecarga (pontuação menor ou igual a 15 pontos), sobrecarga leve (pontuação entre 16 e 22), sobrecarga moderada (pontuação entre 23 e 33) e sobrecarga alta (pontuação maior ou igual a 34)^(21)^.

A HAM-D foi empregada para avaliação da gravidade de sintomas depressivos. O ponto de corte >7 pontos foi utilizado como sugestivo para a presença de sintomatologia depressiva^(22)^.

### Análise dos resultados e estatística

Inicialmente, foi empregado o teste de normalidade de Shapiro Wilk. As variáveis numéricas foram apresentadas por estatística descritiva, sendo calculadas média, mediana e desvio padrão, e as variáveis categóricas foram apresentadas a partir do cálculo das frequências relativa e absoluta.

Como os dados não apresentaram distribuição normal, exceto para a idade do cuidador, o teste de correlação de Spearman foi aplicado para avaliar as correlações entre a variável dependente (sobrecarga do cuidador) e as variáveis independentes, como funcionalidade, fragilidade, cognição, humor, dispneia, fadiga, estresse pós-traumático e fatores sociodemográficos. A sobrecarga do cuidador foi adotada como variável contínua, representada pelo escore total da ZBI, e as variáveis independentes foram selecionadas a partir de revisão na literatura acerca dos sintomas da SPC em pessoas idosas.

A análise de regressão linear foi utilizada para estimar o quanto cada variável independente contribui para variação no escore total da ZBI. Na análise univariada, as variáveis foram avaliadas individualmente, e aquelas com valor de p<0,20 entraram na análise final de regressão linear múltipla, usando o método *backward* para ajustar os possíveis fatores de confusão. Nesse método, todas as variáveis selecionadas foram inseridas, simultaneamente, no modelo, e, em seguida, novos modelos foram estimados por meio de remoção de cada variável com base no maior valor de p.

As análises estatísticas foram realizadas por meio do *software* estatístico *Jeffreys’s Amazing Statistics Program* (*Version* 0.19.1.0). O nível de significância adotado para todas as análises foi de 5% ou p<0,05.

## RESULTADOS

A amostra foi constituída por 75 participantes idosos com SPC e seus respectivos cuidadores, após um período médio de 10,09±5,97 (Mín.=3; Máx.=24) meses entre o diagnóstico de COVID-19 e AV1.

No que diz respeito aos cuidadores de idosos com SPC, o estudo mostrou que 77,92% eram do gênero feminino, com média de idade de 54,99±15,10 anos e 10,75±5,01 anos de escolaridade. Desses, 46,75% eram filhos; 37,66% eram cônjuges; 10,39% eram amigos; e 5,20% eram irmãos. Quanto à renda dos cuidadores, 55,84% não trabalhavam formalmente, e 46,75% recebiam de um a dois salários mínimos. Destaca-se que a maioria (84,62%) dos idosos possuía moradia própria e que 58,44% dos cuidadores residiam com o idoso.

A avaliação pela HAM-D demonstrou que 44,60% dos cuidadores apresentavam sintomatologia depressiva, cuja pontuação média foi de 7,49±5,98 pontos. Entre os deprimidos, 31,51% tinham intensidade leve; 33,51% tinham intensidade moderada; e 34,98% tinham intensidade alta. A sobrecarga do cuidador estava presente em 67% da amostra, sendo que, desses, 33% foram de intensidade moderada, conforme apresentado na Figura 1. A média da ZBI foi de 22,81±13,41 pontos.

**Figura 1:** Prevalência e intensidade da sobrecarga de cuidadores de idosos com síndrome pós-COVID

A presença da sobrecarga do cuidador se manteve constante entre os participantes, mesmo entre aqueles com intervalo de tempo diferente entre o diagnóstico da COVID-19 e a avaliação (p=0,675), conforme mostra a Figura 2.

**Figura 2:** Sobrecarga de cuidadores em relação ao tempo após o diagnóstico de COVID-19 e a data da primeira avaliação

Quanto às características dos idosos que estavam sob cuidado, 51,25% eram do sexo feminino, com média de idade de 73,91±10,20 anos, 8,03±5,78 anos de escolaridade, 1,79±1,79 comorbidades clínicas, polifarmácia (6,54±5,00 medicamentos em uso) e 3,39±1,63 doses da vacina contra COVID-19 no momento da infecção inicial. Além disso, 57,50% foram hospitalizados; 15% passaram por internação em Unidade de Terapia Intensiva; e 70,87% autorrelataram dificuldade de memória após a COVID-19.

Em relação à capacidade cognitiva no momento da primeira avaliação, a maioria (77,5%) dos idosos apresentou alteração cognitiva, sendo 62,5% com comprometimento cognitivo leve, 15%, com demência, e 22,5%, com cognição normal. A avaliação autorrelatada da fragilidade, conforme a CFS prévia (referente a um mês antes do diagnóstico de COVID-19), indicou que 26,25% dos idosos eram frágeis. Na primeira avaliação, observou-se um aumento significativo da fragilidade adquirida após a COVID-19, com 47,50% de idosos frágeis (p<0,001), quando comparada à CFS prévia autorrelatada.

A Tabela 1 mostra a descrição de medidas clínicas, cognitivas, psicológicas e funcionais dos idosos participantes deste estudo:

**Tabela 1:** Descrição de medidas clínicas, cognitivas, psicológicas e funcionais dos idosos avaliados, Belo Horizonte, Minas Gerais, Brasil, 2025

Variável	Instrumento de medida	Média/desvio padrão
Fragilidade prévia	CFS	3,63±1,32
Fragilidade pós-COVID	CFS	4,25±1,36
Fragilidade	IVCF-20	12,93±7,44
Fadiga	FSS	29,68±17,62
Dispneia	mMRC	2,18±1,31
Estresse pós-traumático	PCL-C	29,08±11,26
Depressão do idoso	DSM-5	1,78±2,14
Funcionalidade	ADCS-AVD	63,91±11,21
Funcionalidade	MIF	111,23±11,62
Cognição	AD8	2,89±2,01
Cognição	*Score Z* MDRS	-2,40±3,62

*CFS - Clinical Frailty Scale; IVCF-20 - Índice de Vulnerabilidade Clínico Funcional-20; FSS - Fatigue Severity Scale; mMRC - Modified Medical Research Council; PCL-C - Post-Traumatic Stress Disorder Checklist - Civilian Version; DSM-5 - Diagnostic and Statistical Manual of Mental Disorders - 5^th^ edition; ADCS-AVD - Alzheimer’s Disease Cooperative Study - Activities of Daily Living; MIF - Medida de Independência Funcional; AD8 - Dementia Screening Interview; MDRS - Score Z Mattis Dementia Rating Scale.*

A sobrecarga do cuidador de idosos com SPC apresentou correlação com as seguintes variáveis: sintomatologia depressiva do cuidador, avaliada pela HAM-D (rho=0,523; valor de p<0,001); fragilidade pós-COVID do paciente idoso, medida pelo IVCF-20 (rho=0,347; p=0,002) e CFS (rho=0,385; p<0,001); TEPT do paciente idoso (rho=0,311; p=0,007); sintomatologia depressiva do paciente idoso, medida pelo DSM-5 (rho=0,345; p=0,002); incapacidade funcional do paciente idoso, medida pela ADCS (rho=-0,358; p=0,002) e MIF (rho=-0,364; p=0,001); fragilidade prévia à COVID-19 do paciente idoso, medida pela CFS (rho=0,267; p=0,020); e declínio cognitivo do paciente idoso pós-COVID, avaliado pelo AD8 (rho=0,294; p=0,011).

As variáveis que apresentaram valor de p<0,020 foram selecionadas para análise de regressão linear múltipla. A análise multivariada indicou coeficientes positivos e significativos para a fragilidade do idoso após a COVID-19 (CFS; p=0,022), depressão do idoso (DSM-5; p=0,022) e depressão do cuidador (HAM-D; p<0,001), sugerindo que um aumento nessas variáveis está associado a um aumento na ZBI. Juntos, esses fatores explicam 37% da variância no escore total da ZBI, com R^2^ ajustado=0,370.

## DISCUSSÃO

Os resultados deste estudo indicam que a maioria dos cuidadores de idosos apresenta sobrecarga de intensidade moderada após o diagnóstico de COVID-19. Os principais determinantes foram a fragilidade do idoso após a COVID-19 e a depressão do idoso e do cuidador, que, juntas, foram capazes de explicar 37% da sobrecarga do cuidador do idoso pós-COVID-19, mensurada pela ZBI.

Como resultado da pandemia, cuidadores de idosos, especialmente do sexo feminino, tornaram-se mais predispostos a experimentar um aumento na intensidade da sobrecarga do cuidado e um declínio na autopercepção da saúde^(23)^. Esses dados corroboram os resultados do atual estudo, bem como de outro estudo brasileiro publicado em 2022, que descreve a sobrecarga, sintomatologia depressiva e o perfil de cuidadores informais de idosos na pandemia de COVID-19^(24)^.

Estudo publicado em 2022, que explorou fatores associados ao aumento da sobrecarga do cuidador durante a pandemia, observou a associação entre sintomas depressivos nos cuidadores e escores mais elevados de sobrecarga de cuidados (OR=2,20; IC95%, 1,50-3,23), resultado similar ao do presente estudo^(6)^. Além da sintomatologia depressiva do cuidador, outros fatores também foram associados com a sobrecarga do cuidador, como baixos escores do Índice de Barthel (OR=2,01, IC95%, 1,39-2,90), presença do diagnóstico de demência (OR=2,48, IC95%, 1,07-5,73), tempo de cuidado (OR=1,09, IC95%, 1,01-1,17) e uso de serviço de atenção domiciliar (OR=1.46; IC95%, 1,01-2,10)^(7)^. Porém, divergem dos nossos, provavelmente, em razão das características da amostra, da diferença nos instrumentos de avaliação utilizados, e do tempo entre o diagnóstico e a avaliação.

Outro estudo, publicado em 2021, investigou fatores associados à sintomatologia depressiva em cuidadores de idosos com diagnóstico de COVID-19 durante a pandemia na Sérvia, e encontrou resultados análogos a este estudo^(25)^. Os autores observaram que 71,9% dos cuidadores informais experimentaram sobrecarga de cuidados e 27,1% deles apresentaram sintomatologia depressiva. A autoavaliação da saúde física, a necessidade de apoio psicossocial e a sobrecarga do cuidador foram os principais preditores diretos da depressão do cuidador. Os autores sugerem que a sobrecarga autorrelatada seja um fator de risco significativo para sintomas depressivos em cuidadores de idoso com COVID-19 durante a pandemia^(25)^.

Cuidar de um idoso frágil é um processo dinâmico que também pode fragilizar o cuidador, por se sentir impotente diante de necessidades abrangentes, uma vez que a fragilidade do idoso se associa, geralmente, a condições clínicas desfavoráveis, tais como suscetibilidade a doenças, declínio funcional, quedas, hospitalização e morte precoce^(26)^. Além disso, o isolamento social prolongado, intensificado pela pandemia, somado à presença de múltiplas doenças crônicas, pode exacerbar problemas de saúde mental, como depressão e ansiedade, no idoso^(6,9,24)^.

Adicionalmente, cuidadores de idosos são reconhecidos como um grupo psicologicamente vulnerável, especialmente quando suas próprias necessidades são negligenciadas^(25)^. A pandemia agravou as limitações no acesso a serviços formais de saúde, já caracterizados por insuficiência estrutural e de cobertura, impondo aos cuidadores informais a necessidade de assumir responsabilidades adicionais em condições mais adversas^(25)^. Essa combinação de fatores exige que os cuidadores forneçam assistência constante, o que pode gerar um estresse excessivo, tanto físico quanto emocional^(6,9,24)^.

Estudo realizado em Minas Gerais, em 2022, mostrou que, durante a pandemia, os idosos evoluíram com maior dependência em atividades instrumentais de vida diária, bem como de piora da cognição^(27)^, o que foi acompanhado por uma diminuição da disponibilidade das famílias para atender a essa nova demanda^(28,29)^.

Apesar de estudos prévios abordarem fatores adicionais, como a incapacidade cognitiva e funcional, associados à sobrecarga do cuidador familiar do idoso durante a pandemia de COVID e de idosos na comunidade^(30,31)^, no presente estudo, não observamos correlação dessas variáveis com a sobrecarga dos cuidadores de idosos após infecção por COVID-19 na análise multivariada. Uma justificativa plausível seria que os sintomas persistentes da SPC impactam, possivelmente, as atividades de vida diária mais complexas, como as instrumentais e avançadas, o que implica menor demanda de cuidado físico para os cuidadores familiares, quando comparado ao acometimento de atividades básicas de vida diária^(32)^. Há que se enfatizar que essas atividades, especialmente a participação social, evidenciam um processo de envelhecimento bem-sucedido^(33)^, e são precursoras de um declínio adicional futuro em atividades de menor complexidade cognitiva, como as de autocuidado^(34)^. Além disso, as ações de cuidado a longo prazo se tornam repetitivas, podendo levar o cuidador a desenvolver estratégias adaptativas, como buscar ajuda de outros indivíduos, tanto para desenvolver as atividades de cuidado requeridas pelo idoso quanto para si próprio, especialmente para se desvencilhar temporariamente desse papel^(28,31)^.

Evidências sugerem que o cuidado de um paciente frágil, deprimido e dependente após o diagnóstico de COVID-19 pode ser uma experiência traumática e negativa para o cuidador, levando ao desenvolvimento de estresse emocional e aumento da intensidade da sobrecarga, uma vez que o cuidador precisa assumir gradativamente mais tarefas a longo prazo^(5,24)^.

Por fim, os resultados deste estudo apontam para a necessidade inadiável de ampliação e fortalecimento de políticas públicas voltadas à população idosa e seus cuidadores, após o diagnóstico de COVID-19, nos seus diferentes âmbitos de assistência^(29)^.

Destaca-se a necessidade de estudos futuros, especialmente estudos longitudinais, para estabelecer fatores de risco e avaliar a evolução da sobrecarga de cuidados de idosos com SPC.

### Limitações do estudo

Este estudo apresenta algumas limitações, tais como a amostra unicêntrica e realizada em sua maioria com idosos previamente hospitalizados por COVID-19, o que pode dificultar a generalização dos resultados. O estudo não apresenta um grupo controle, e há ausência de dados prévios à COVID-19, especialmente da presença de sobrecarga, que não foi avaliada retrospectivamente, a fim de evitar um viés de memória dos participantes, em virtude de seu caráter subjetivo. Além disso, não foi avaliado o tempo de permanência do cuidador informal junto ao idoso, o que pode influenciar, ao menos em parte, os resultados de sobrecarga encontrados. Este trabalho, embora utilize medidas padronizadas, apresenta questionários autorrelatados que podem estar sujeitos a viés de memória e informação, relacionados às condições físicas e psicológicas do idoso e do cuidador. Adicionalmente, observou-se um aumento significativo da fragilidade adquirida após a COVID-19. Entretanto, o desenvolvimento de morbidades, reinternações hospitalares e outras intercorrências no período entre o diagnóstico de COVID-19 e a aplicação do protocolo pode ter contribuído para o aumento da fragilidade na população estudada.

Por fim, o desenho deste estudo não permite a identificação de fatores de risco, e pode não ter analisado outros fatores que, possivelmente, poderiam estar relacionados à sobrecarga do cuidado nessa população.

### Contribuições para a área da saúde

Este é um dos poucos estudos que se propôs a investigar, por meio de avaliação geriátrica ampla e presencial, a prevalência e fatores associados à sobrecarga de cuidadores de idosos com SPC. Os resultados deste trabalho podem ajudar a direcionar as intervenções da equipe interdisciplinar para o cuidado dessa população, uma vez que identifica fatores passíveis de medidas de prevenção, controle e reabilitação.

## CONCLUSÕES

Os achados deste estudo demonstram a ocorrência de sobrecarga em cuidadores de idosos com SPC. Essa condição foi de intensidade moderada, associando-se positivamente à fragilidade do idoso e à sintomatologia depressiva do idoso e do cuidador.

Ao identificar a ocorrência e os fatores associados à sobrecarga de cuidadores de idosos com SPC, o presente estudo traz informações significativas e tem implicações importantes para a prática clínica, pois revela indicadores, sugerindo que os profissionais de saúde devem estar atentos ao binômio cuidador-idoso.

## Data Availability

Os dados de pesquisa só estão disponíveis mediante solicitação.
